# Surveillance and Selective Treatment of *Brugia malayi* Filariasis Eleven Years after Stopping Mass Drug Administration in Belitung District, Indonesia

**DOI:** 10.4269/ajtmh.23-0255

**Published:** 2023-11-27

**Authors:** Taniawati Supali, Yenny Djuardi, Santoso, Lita Renata Sianipar, Nungki Hapsari Suryaningtyas, Rahmat Alfian, Yossi Destani, Elisa Iskandar, Hendri Astuty, Noviani Sugianto, Peter U. Fischer

**Affiliations:** ^1^ Department of Parasitology, Faculty of Medicine, Universitas Indonesia, Jakarta, Indonesia;; ^2^ Baturaja Unit for Health Research and Development, National Institute of Health Research and Development, Ministry of Health of Indonesia, South Sumatra, Indonesia;; ^3^ Directorate of Communicable Disease, Prevention, and Control, Indonesia Ministry of Health, Jakarta, Indonesia;; ^4^ Infectious Diseases Division, Department of Medicine, Washington University School of Medicine, St. Louis, Missouri

## Abstract

*Brugia malayi* is the major cause of lymphatic filariasis (LF) in Indonesia. Zoophilic *B. malayi* was endemic in Belitung district, and mass drug administration (MDA) with diethylcarbamazine (DEC) and albendazole ceased after five annual rounds in 2010. The district passed three transmission assessment surveys (TAS) between 2011 and 2016. As part of the post-TAS3 surveillance of the national LF elimination program, we collected night blood samples for microfilaria (Mf) detection from 1,911 subjects more than 5 years of age in seven villages. A *B. malayi* Mf prevalence ranging from 1.7% to 5.9% was detected in five villages. Only 2 (5%) of the total 40 Mf-positive subjects were adolescents aged 18 and 19 years old, and 38 (95%) Mf-positive subjects were 21 years and older. Microfilarial densities in infected individuals were mostly low, with 60% of the subjects having Mf densities between 16 and 160 Mf/mL. Triple-drug treatment with ivermectin, DEC, and albendazole (IDA) was given to 36 eligible Mf-positive subjects. Adverse events were mostly mild, and treatment was well tolerated. One year later, 35 of the treated Mf-positive subjects were reexamined, and 33 (94%) had cleared all Mf, while the anti-Bm14 antibody prevalence remained almost unchanged. Results indicate that in *B. malayi*-endemic areas, post-TAS3 surveillance for Mf in the community may be needed to detect a potential parasite reservoir in adults. Selective treatment with IDA is highly effective in clearing *B. malayi* Mf and should be used to increase the prospects for LF elimination if MDA is reintroduced.

## INTRODUCTION

The Global Program to Eliminate Lymphatic Filariasis is based mainly on mass drug administration (MDA) using microfilaricidal drugs. Indonesia joined the program in 2002 and uses mostly diethylcarbamazine (DEC) plus albendazole for MDA. A total of 236 of the 514 districts in Indonesia have been identified as requiring MDA for lymphatic filariasis (LF).^1^ Belitung district was classified as endemic for LF in 2004 based on a *Brugia malayi* microfilaria (Mf) rate of 3.4% in Sual Gual village (Ministry of Health, unpublished data). The district was one of the first adopters of annual MDA using a single dose of DEC plus albendazole in Indonesia, and five rounds were given from 2006 to 2010 to the entire eligible population. The reported treatment coverages of eligible population for the five rounds of MDA were 83.9%, 73.9%, 81.9%, 86.1%, and 89.1%, respectively (Ministry of Health, unpublished data). Since treatment coverage was constantly above 65%, pre-transmission assessment surveys (pre-TAS) and TAS were performed according to the guidelines of the WHO.^2^

The WHO guidelines for monitoring and evaluating the LF elimination program recommends the use of the Brugia Rapid^TM^ test to detect IgG4 antibodies reactive with the recombinant BmR1 antigen in schoolchildren of grades 1 and 2 in all areas endemic for brugian filariasis.^2^ After a pre-TAS performed in a convenient sample of children and adults living in two high-risk sites per evaluation unit is passed, three TAS are recommended, ideally 2 years apart. When all TAS are passed, the evaluation unit can be approved to enter the “post-TAS3” or postelimination surveillance stage. In Belitung district, pre-TAS using Mf detection in night blood was passed in 2010 with no Mf-positive subjects. In the following year, TAS1 was passed with zero out of 1,433 schoolchildren positive for filarial IgG4 antibodies. TAS2 was performed in 2014 and found two antibody-positive children (*N* = 1,522), and finally, TAS3 done in 2016 showed no antibody-positive schoolchildren (*N* = 1,590). Based on these results, the Belitung district evaluation unit was nationally classified as LF-free in 2017 by the Indonesian Ministry of Health.^3^ A post-TAS3 surveillance survey to reiterate the conclusions from the TAS surveys using an alternative diagnostic method with the entire community as the target population was initiated by the National Institute of Health Research and Development (Ministry of Health). Three years after the last TAS, a night blood survey in the initial village used for mapping (Sual Gual) and a cross-check site (Lassar, a village with reported clinical cases of lymphedema) was conducted. The results from this small-scale survey were unexpected and alarming: in the villages of Sual Gual and Lassar, Mf prevalences of 2.2% and 5.1%, respectively, were detected. Subsequently, the Mf-positive subjects received individual treatment with DEC and albendazole; however, broad-scale intervention was not feasible at that time. Although high MDA coverage was reported from Belitung district, a compliance survey by convenience sampling of 526 subjects in the villages of Sual Gual and Lassar using a special questionnaire indicated a rate of compliance to previous treatment of only 60%.^4^

As part of the national LF elimination program, we performed in 2021 independent surveys to confirm the Mf results from the two villages and to increase the sampling areas. Night blood Mf surveys were conducted in seven villages located in four of the five subdistricts of Belitung. Our results show that *B. malayi* is still endemic in Belitung and that further intervention efforts are required. Selective treatment of 36 Mf-positive subjects showed that triple-drug treatment using ivermectin, DEC, and albendazole (IDA) is highly effective in clearing Mf of *B. malayi*.

## MATERIALS AND METHODS

The surveillance study was approved by the ethics committee of the Faculty of Medicine, Universitas Indonesia, Jakarta (number KET-650/UN2.F1/ETIK/PPM.00.02/2021).

### Study area.

Belitung district is located on Belitung island, with its adjacent island close to the southeastern tip of the island of Sumatra. The district is divided into five subdistricts, Tanjung Pandan, Membalong, Badau, Sijuk, and Selat Nasik (located on Mendanau island), with a total of 49 villages. In 2021, it was reported that the total population was 193,493 people, consisting of 100,406 males and 93,087 females.^5^ About half of the total population lives in the Tanjung Pandan subdistrict, where the capital city is located.^5^ Belitung district is known for producing white pepper and tin, but the residents recently switched to palm oil production because of government regulations forbidding residents from private mining. Many mining pits were left open after cessation of mining and could become potential breading sites for mosquitoes.

**Figure 1.**
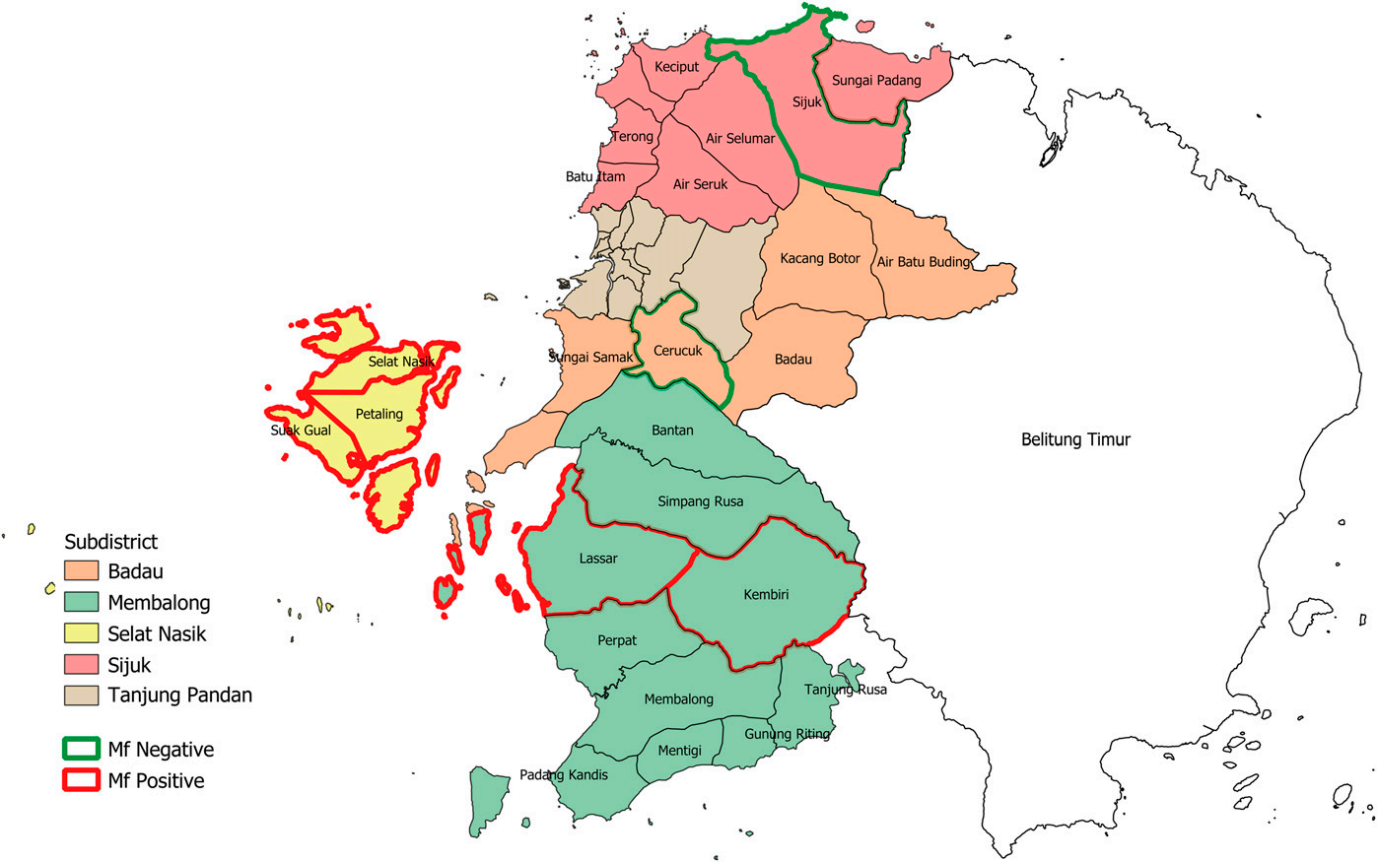
Distribution of study villages (desa) in Belitung district.

Seven villages were selected for this study based on their history of LF and geographic location to cover all subdistricts except the capital city of Tanjung Pandan. Three villages (Selat Nasik, Sual Gual, and Petaling) were located in the Selat Nasik subdistrict on Mendanau island, two villages (Lassar and Kembiri) were located in the Membalong subdistrict, and one village was located in each of the Badau (Cerucuk) and Sijuk (Sijuk) subdistricts (Figure 1).

The survey was conducted in June 2021, during the COVID-19 pandemic, and special precautions were taken during the survey. Recruitment of study participants was done either in or near the village hall or in or near the village head’s house. No more than 50 persons were gathered in a single night, and social distancing rules were followed. The study team was fully vaccinated against COVID-19 according to the definition of the Indonesian Ministry of Health. The members of the research team wore personal protective equipment and distributed surgical masks to the study participants.

After the purpose of the study was explained to the communities, individuals were asked for verbal informed consent to participate in the study, as written consent is not in agreement with the local sociocultural habits. For children below the age of 17 years, verbal informed consent was obtained from their parents or relatives. Originally, a convenience sample of 200 subjects per village was planned with an oversampling of 1,000 subjects in one large high-risk village (Lassar). Because the study was performed during the COVID-19 pandemic, this was not always feasible if COVID-19 cases were identified. Study participants were registered by their name, age, and sex. To minimize the risk of COVID-19 transmission, identical study teams visited each village. The study team consisted of a small number of persons: one nurse or technician from the primary health center (Puskesmas), the head of the village, one or two cadres/health staffs from the district, and one or two members of the Universitas Indonesia research team. The study team enrolled and collected blood samples from 50 subjects per night and worked in each location for 3–4 days.

### Microscopic examination.

A finger prick of a night blood sample (250 μL) from each participant was collected in an ethylenediaminetetraacetic acid-coated Microtainer tube between 8:00 pm and 12:00 am. Three-line thick blood smears were prepared, dried for 2 days, and stained with Giemsa as described previously.^6^ The blood slide was examined under the microscope by two experienced microscopists to detect, specify, and count the Mf. The rest of the samples were stored at –20°C for antibody testing using the Bm14 antigen.

### Ivermectin, DEC, and albendazole treatment.

Triple-drug treatment with ivermectin, DEC, and albendazole was administered to eligible, Mf-positive subjects based on the guidelines developed by the Indonesian Ministry of Health in accordance with the matching WHO guidelines.^7^^,^^8^ The treatment was given by health district and primary health center staffs. Adverse events were observed for up to 48 hours after treatment and recorded.

### Measurement of antifilarial IgG4 antibodies before and after treatment in Mf-positive subjects.

At the time of the study, the WHO did not recommend the use of the Brugia Rapid test, which uses the BmR1 antigen to measure antifilarial IgG4, because of quality control problems. Therefore, we used an in-house indirect ELISA that uses recombinant Bm14 protein as the antigen as described previously.^9^ Antibody testing was performed on plasma samples of microfilaremic participants. A pooled plasma sample from four subjects infected with *Brugia timori* (Mf density between 70 and 1,111 Mf/mL) was used as a positive control. Plasma samples taken from 13 healthy volunteers residing in nonendemic areas in Indonesia were used as a negative control. The cutoff value was obtained by determining the average (mean) optical density (OD) of negative samples ± 3 SD. When the OD of sample measurement was above the cutoff value, it was considered positive. The cutoff value for Bm14-specific IgG4 positivity was taken from the mean OD value of 13 negative controls plus 3 SD, which was 0.014.

### Data analysis.

Data such as age, sex, village, and Mf count were entered into an Excel program and transferred to SPSS v. 20 (IBM., Armonk, NY) for statistical analysis. The χ^2^ test was used to analyze the Mf rate between male and female participants as well as the difference in prevalence among age groups. A comparison of Bm14-specific IgG4 levels before and after treatment was performed using the Wilcoxon signed-rank test. All statistical analyses were performed in IBM SPSS Statistics v. 20.

## RESULTS

### Characteristics of study population.

A total of 1,911 persons participated in the surveillance study. Among these participants were 529 subjects from the Selat Nasik subdistrict of Mendanau island, and 1,382 persons were from three subdistricts, Membalong, Badau, and Sijuk, of the main island of Belitung. The study focused on these villages because, historically, microfilaremic and elephantiasis cases were reported by the primary health care center in these subdistricts. Most participants were above 50 years of age (24.3%), and only 15.1% were under the age of 21 years (Table 1). In all age groups, about 10% more female subjects than male subjects participated in the study.

**Table 1:** Age and sex distribution of study participants

Age group (years)	Females	Males	Totals
6–20	150	139	289
21–30	186	155	341
31–40	244	192	436
41–50	219	161	380
≥ 51	252	213	465
Total	1,051	860	1,911

### Prevalence of microfilaremia.

Prevalence was determined by detection of Mf in night blood smears. Microscopic examination identified 40 microfilaremic participants. All detected Mf were unambiguously identified as *Brugia malayi* by morphology. No subjects with *Wuchereria bancrofti* Mf were recorded. Mf-positive subjects were found in five of the surveyed seven villages, but the sample size for the two Mf-negative villages was the lowest (Figure 1). In the five positive villages, Mf rates ranged from 1.7% to 5.9%, with an overall Mf rate of 2.1% (Table 2). The highest prevalence of Mf was observed in the Selat Nasik subdistrict, Mendanau island, where *B. malayi* was endemic in all three study villages, with Mf rates of 1.8%, 1.5%, and 5.9%.

**Table 2:** Prevalence of subjects with *B. malayi* microfilaremia by study village

Subdistrict	Village	No. positive/total no. (Mf rate)		
		Females	Males	Total
Selat Nasik*	Selat Nasik	1/177 (0.6%)	4/99 (4.0%)	5/276 (1.8%)
	Sual Gual	1/103 (1.0%)	2/99 (2.0%)	3/202 (1.5%)
	Petaling	2/29 (6.9%)	1/22 (4.6%)	3/51 (5.9%)
Membalong†	Kembiri	1/95 (1.1%)	2/79 (2.5%)	3/174 (1.7%)
	Lassar	8/588 (1.4%)	18/501 (3.6%)	26/1,089 (2.4%)
Badau†	Cerucuk	0/38 (0%)	0/40 (0%)	0/78 (0%)
Sijuk†	Sijuk	0/21 (0%)	0/20 (0%)	0/41 (0%)
Total		13/1,051 1.2%	27/860 (3.1%)	40/1,911 (2.1%)

Mf = microfilaria.

* Located on Mendanau island.

† Located on Belitung island.

On the main island of Belitung, *B. malayi* was endemic in both villages of the Membalong subdistrict, Lassar and Kembiri. In Lassar village alone, where 1,089 blood samples were assessed, 26 Mf-positive subjects were found. It had been reported that in 2019 this village has a Mf rate of 5.1%.^4^ Lassar village was divided into six subvillages, and samples were collected in each subvillage. The Mf rates in the subvillages ranged from 1.1% to 3.3%, indicating that *B. malayi* was endemic in all parts of the village. Although more female than male subjects were tested, the Mf rate was significantly higher in males than females (3.1% versus 1.2%; chi-square test, *P* = 0.004).

When all study populations were categorized based on age groups, the percentage of Mf-positive subjects increased with age (chi-square test, *P* < 0.0001). There were two boys aged 6 and 10 years old participating in the blood collection. Both of them were Mf negative. Most examined children and teenagers were above the age of 10 years. The youngest microfilaremic subjects were two males, aged 18 and 19 years old, with Mf counts of 128 and 784 Mf/mL, respectively. Both subjects were from Lassar village. The highest rate of Mf positives was in the age group above 50 years (Table 3).

**Table 3:** *B. malayi* Mf prevalence classified by age group

Age group (years)	Mf positive (*n*/*N*)	Mf rate (%, CI)
6–20	2/289	0.7 (0–1.66)
21–30	3/341	0.9 (0–1.87)
31–40	5/436	1.1 (0.15–2.15)
41–50	9/380	2.4 (0.84–3.90)
≥ 51	21/465	4.5 (2.63–6.40)

Mf = microfilaria.

### Microfilarial density.

The Mf density of the 40 microfilaremics ranged from 16 to 6,064 Mf/mL. A total of 24 microfilaremic participants (60%) had low Mf densities, ranging from 16 to 160 Mf/mL, 11 participants had Mf densities of 161 Mf/mL to 800 Mf/mL, and two participants had Mf densities between 801 Mf/mL and 1,600 Mf/mL. Three participants from Lassar village, one female (1,904 Mf/mL) and two males (2,320 and 6,064 Mf/mL), had high Mf densities.

### Selective treatment with ivermectin, DEC, and albendazole: adverse events.

IDA treatment was given to 36 of 40 microfilaremic individuals. Treatment was postponed for four persons due to hypertension (Indonesian Ministry of Health recommendation) and/or pregnancy. Seven individuals (19.4%) reported adverse events, ranging from a minimum of one symptom (one person) to a maximum of five symptoms (one person). Five persons reported three symptoms, including one person with the highest Mf density of 6,064 Mf/mL. Fever was experienced by all individuals with adverse events, followed by headache (six persons), myalgia or arthralgia (four persons), nausea (three persons), and vomiting (one person). The participant who reported five symptoms, which lasted for 18 hours, had a Mf density of 784 Mf/mL. The other participants had adverse events with a duration not longer than 10 hours after the first report.

### Comparison of Mf densities before and after treatment.

**Figure 2.**
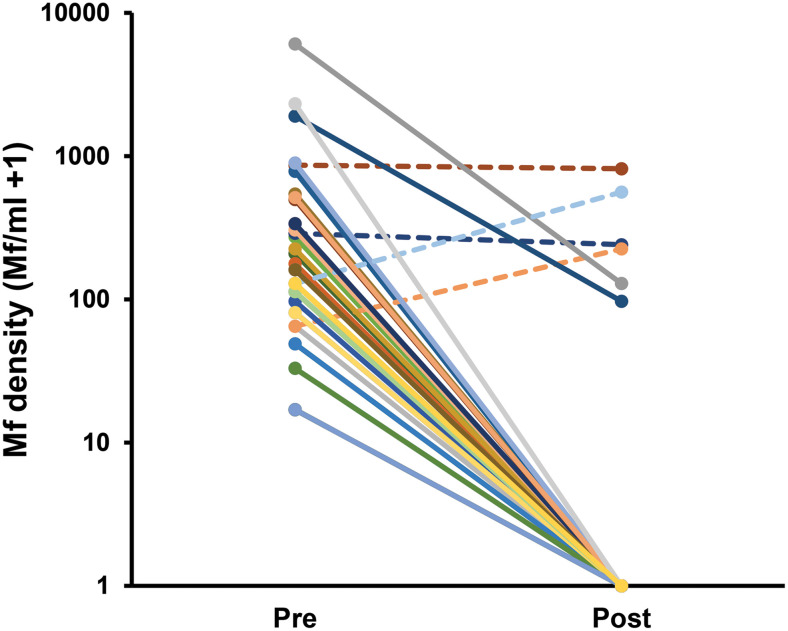
Microfilarial (Mf) density (logarithmic scale) of subjects infected with *B. malayi* in 2021 before (pre) any treatment and in 2022 after (post) treatment with albendazole (solid lines). Broken lines indicate Mf densities of subjects that were not eligible for treatment.

At 1 year post-IDA treatment, one Mf-positive subject at baseline refused to participate in the night blood collection; therefore, this subject was excluded from further analysis. Thirty-three (94%) of 35 treated patients became Mf negative during the follow-up period. The remaining two Mf-positive patients were still Mf positive after IDA treatment, although the Mf density decreased in comparison with that during pretreatment (from 1,904 to 96 Mf/mL and from 6,064 to 128 Mf/mL). On the other hand, the four untreated patients remained Mf positive with stable or increased Mf densities (Figure 2).

### Prevalence of IgG4 antibody in Mf-positive patients.

Paired blood samples (before and after IDA) from 34 treated subjects were available to be tested for the presence of IgG4 antibodies against Bm14 antigen. The posttreatment plasma sample of one treated subject was not sufficient for antibody testing, and therefore this individual was excluded from the analysis. The two treated participants with persistent Mf positivity 1 year later had no significant change in Bm14-specific IgG4 levels (*P* = 0.180) (Table 4). On the other hand, most microfilaremics (32 individuals) who became negative after treatment had a significant decrease (*P* = 0.011) in Bm14-specific IgG4 levels from pretreatment (median OD, 1.57; interquartile range [IQR], 0.34–2.96) to posttreatment (median OD, 1.17; IQR, 0.13–2.55). One microfilaremic male (32 years old) from Lassar village who had been previously positive for Bm14-specific IgG4 and became negative for both microfilaremia and antibody levels had an already low level of Bm14-specific IgG4 (OD, 0.035) although still above the cutoff. Interestingly, there was a microfilaremic female (57 years old) with a density of 80 Mf/mL from Selat Nasik village who was negative for Bm14-specific IgG4 before and after treatment.

**Table 4:** Levels of Bm14-specific IgG4 based on Mf status before and after treatment

Mf status pre-Tx	Mf status post-Tx	Bm14-specific IgG4 pre-Tx		Bm14-specific IgG4 post-Tx	
		Positive (*n*)	Negative (*n*)	Positive (*n*)	Negative (*n*)
Positive	Positive	2	0	2	0
	Median OD (IQR)	2.08 (NA)		2.22 (NA)	
	Negative	31	1	30	2
	Median OD (IQR)	1.61 (0.48–3.00)	0.0025 (NA)	1.32 (0.17–2.69)	0.0025 (0.0025–0.0026)
Total		33	1	32	2

IQR = interquartile range; Mf = microfilaria; NA = not applicable; OD = optical density; post-Tx = after treatment; pre-Tx = before treatment.

## DISCUSSION

This post-TAS3 survey confirmed that LF caused by *B. malayi* is endemic in several villages of Belitung district 11 years after cessation of MDA with DEC plus albendazole. A previous survey conducted 3 years after the TAS3 observed *B. malayi* microfilaremia in two villages, and the present study added another three villages with microfilaremia.^4^ Since the reintroduction of MDA in areas that have passed TAS3 is a major logistic and financial investment, confirmatory surveillance surveys are important before any decision-making. Selective treatment of Mf-positive subjects or MDA in a few villages where *B. malayi* is endemic may be sufficient and more cost-effective than reintroduction of MDA in the entire evaluation unit. Unfortunately, there is little guidance available for countries of endemicity regarding post-TAS3 surveillance design and response to positive surveillance results. Heavily clustered infection that is limited to a small number of villages may justify the redefinition of implementation units for MDA and help to save resources.

No children under the age of 16 years were positive for *B. malayi* Mf, and most subjects with microfilaremia were older adults who were not surveyed during TAS. Therefore, it is difficult to decide whether microfilaremia in adults was always present and the TAS strategy that uses antibody assays in school-aged children was not sensitive enough to detect ongoing transmission or if adults were preferentially exposed to zoophilic *B. malayi* from a potential animal reservoir and got reinfected. In any case, our observation that Mf positivity was exclusively found in adults may support post-TAS3 surveillance only in this part of the population. This target group will help to increase the sensitivity of surveillance surveys. The Ministry of Health had reported that between the years 2006 and 2010, Belitung district received MDA with a treatment coverage of over 65%.^1^ However, it is impossible to check now how accurate the coverage reporting was and whether low treatment coverage in adults contributed to the failure to permanently eliminate LF in the district. It is also possible that there was a substantial gap between MDA coverage and compliance and that local health officers lacked knowledge to properly calculate compliance or drug ingestion.^10^ A study in the Agam and Depok districts of Indonesia showed that there is a coverage-compliance gap and that not all local populations react the same way to MDA campaigns, underlining the importance of proper health educational materials to improve treatment compliance.^11^ In some areas, specific compliance surveys can be very helpful to assess the strength of the MDA program. This is especially important in areas with IDA MDA, since fewer rounds are needed and higher compliance in each round is needed for effective intervention.

More females than males participated in our surveys, but the number of Mf positives was higher in males, which is a common finding in Indonesia.^6^^,^^12^ The observation that the prevalence of *Brugia* is higher in males than in females has several explanations. In areas with brugian filariasis, men are often more frequently exposed to outdoor biting mosquitoes that transmit the infective larvae. It was reported in 1989 that *Mansonia* species are the main vectors for *B. malayi* on Sumatra, which is the largest island close to Belitung island, and that this species is suspected to also be the vector on Belitung.^13^ Ponds that serve as the breeding places for *Mansonia* are found scattered near all endemic villages on Belitung but not in the district capital of Tanjung Pandan and adjacent areas.

The study area easily passed TAS1 and two more TAS leading into the post-TAS3 phase for which no official guidance of the WHO on surveillance is currently available.^1^ One analysis of different evaluation units showed that failure to pass TAS is associated with brugian filariasis,^14^ while another study showed no association between *Brugia* endemicity and the probability of passing pre-TAS.^15^ Both studies included evaluation units in Indonesia, but a major difference was that TAS used the Brugia Rapid^®^ antibody test in school-aged children as a diagnostic tool whereas for pre-TAS, Mf detection in night blood samples from the community was used. Our study supported the urgency of further post-TAS3 surveillance for the population at the highest risk of infection, which in this case for Belitung is adults rather than children. Rao et al.^16^ conducted a study in Sri Lanka that showed also for bancroftian filariasis that passing the three TAS in schoolchildren may not be a sufficient indicator of complete elimination of LF.

In the present study, the levels of Bm-14 specific antibody were decreasing in all treated individuals who became amicrofilaremic, except for the microfilaremic individuals who had not received treatment due to various health reasons. Until now, serology testing for brugian filariasis has relied only on detection of a specific antibody, and for many years, a rapid test format (Brugia Rapid) was used to implement the TAS. Recently, another study done in an eastern part of Indonesia has shown that Bm14-specific IgG4 measurement by ELISA could be an alternative to the Brugia Rapid test to evaluate the response to treatment when microfilaremia is no longer detected.^9^ Although this assay could not differentiate between present and past infections, the availability of longitudinal data on antibody levels before and after MDA could help determine whether the infection has persisted.^17^ One microfilaremic person consistently tested negative for Bm-14 specific IgG4 before and after treatment. This phenomenon was also found in a previous study in an area in Indonesia where *B. timori* was endemic,^9^ as well as in an area in Haiti where *W. bancrofti* was endemic.^18^ Several explanations can be offered. First, it is possible that some microfilaremic individuals do not produce IgG4 but rather another antibody isotype such as IgG1.^19^ Another possibility is that certain microfilaremic individuals may have immune unresponsiveness against filarial antigens,^20^ although not necessarily accompanied by high Mf density.

Triple-drug treatment using IDA has been shown to be highly efficient for long-term clearance of microfilaremia caused by *W. bancrofti* or *B. timori*.^21^^–^^23^ As a result, this treatment has been recommended by the WHO as an alternative MDA strategy to speed up elimination.^8^ IDA has been used in hot spot areas in Malaysia where *B. malayi* is endemic, but the efficacy of IDA for *B. malayi* has not yet been formally tested.^24^ Therefore, this present report fills an important gap, as it shows that IDA is also safe and highly efficacious (94% Mf clearance) in subjects infected with *B. malayi*. This result is in line with previous results of our group that showed 96% Mf clearance after 1 year for the closely related species *B. timori*.^23^

In conclusion, our study showed that LF still exists in certain areas in Indonesia despite five annual rounds of MDA with DEC and albendazole, sufficient reported coverage, and three passed TAS. Post-TAS3 surveillance specifically targeting the population most likely to be at risk of infection with *B. malayi* is crucial to achieve the goal of permanent elimination. Based on these results, the Ministry of Health of Indonesia quickly reacted by performing extensive baseline surveys in the entire district using the WHO-recommended IDA impact survey design and restarted MDA. Together with an improved public health campaign to ensure strong compliance, IDA treatment has been introduced to Belitung.
